# Prostate abscess in a paediatric patient: A rare diagnosis

**DOI:** 10.4102/sajr.v25i1.2053

**Published:** 2021-03-25

**Authors:** Atish Vanmali, Mario Haines

**Affiliations:** 1 Department of Radiology, Jackpersad and Partners Inc., Durban, South Africa

**Keywords:** paediatric, prostate, abscess, obstructive uropathy, paediatric imaging

## Abstract

Prostate abscess (PA) is an uncommon clinical manifestation that typically presents in the fifth to sixth decade of age and sporadically affects neonates. These characteristics, coupled with the atypical presentation, represent a clinical dilemma and a challenging diagnosis. A detailed history depicting the clinical course and the presence of risk factors is imperative to alert the clinician of the possibility of a PA. In this case report, we present a surgically confirmed PA, a diagnosis that is rarely encountered within the paediatric age group.

## Introduction

Prostate abscess (PA) occurs as a result of the focal accumulation of pus in the prostate gland and is commonly related to the spread of Gram-negative bacilli from the urinary tracts.^1^ W. Allison in 1842 initially described a fatal case of a PA complicated by spontaneous urethral rupture into the recto prostatic fascia.^2^

Prior to the use of antibiotics, gonorrhoeal infection was the most prevalent aetiology. However, with the advent of antibiotic use, Gram-negative bacteria are the most common organisms encountered and are responsible for 60% – 80% of cases.^3^ New at-risk populations and the wide range of local and systemic symptoms present a clinical challenge in diagnosis.^3^

## Case report

A 6-year-old male with a medical history of post streptococcal glomerulonephritis presented to the hospital with clinical symptoms of headache and pelvic pain for 2 days.

**FIGURE 1:**
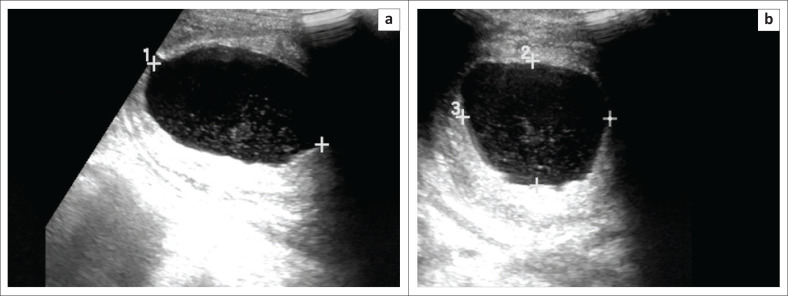
Ultrasound images of the bladder in the sagittal planes (a) and transverse (b) indicate multiple intravesical low-level echoes. These were mobile on real time B-mode scan.

**FIGURE 2:**
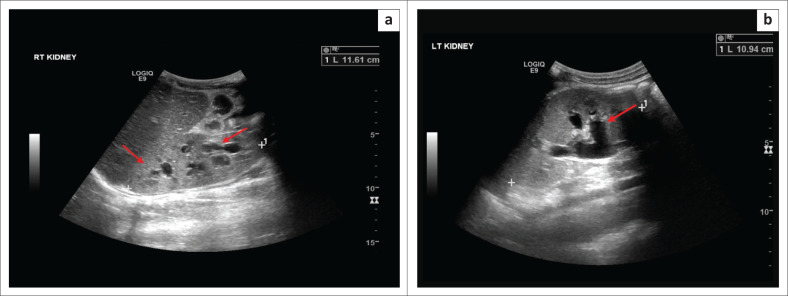
Ultrasound of the right kidney (a) and left kidney (b) demonstrating bilateral hydronephrosis and enlarged kidneys.

Haematuria was noted in his urinalysis. The patient’s symptoms persisted over 2 days progressing to fever and suprapubic pain. An ultrasound of the lower renal tracts (Figure 1) revealed a bladder volume of 82.05 millilitres (mL), bladder wall thickening and fine mobile echoes within the bladder. The upper renal tracts demonstrated bilateral hydronephrosis (Figure 2) and bilateral renal enlargement (right kidney 11.61 centimetres [cm], left kidney 10.94 cm). Minimal free fluid was noted in the pelvis; however, no prostatic lesion was appreciated on the transabdominal ultrasound.

**FIGURE 3:**
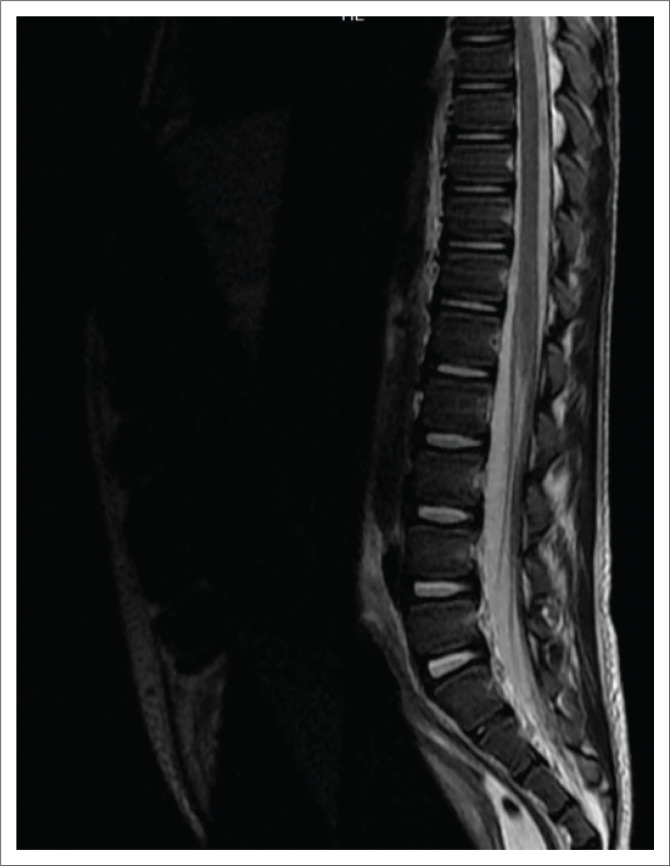
Sagittal T2-weighted magnetic resonance imaging sequence of the lower thoracic and lumbar spine demonstrating normal signal intensity in the spinal cord and conus medullaris.

**FIGURE 4:**
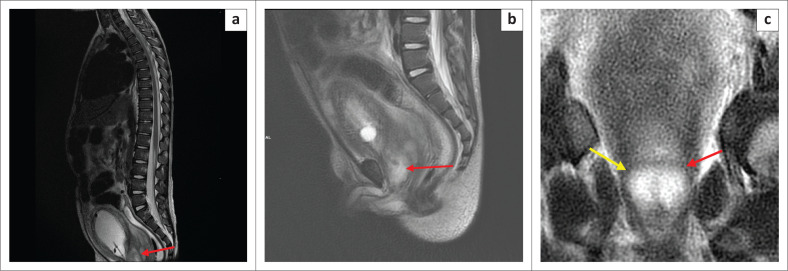
Magnetic resonance imaging: Sagittal T2-weighted sequence (a) of the thoracic and lumbar spine. Incidental cyst of the prostate gland was noted. Sagittal and coronal T2-weighted sequences (b and c, respectively) of the pelvis demonstrate a well-defined homogenous cystic lesion within the prostate gland in keeping with a prostatic cyst.

The voiding cysto-urethrogram was normal with no features of vesicoureteral reflux or posterior urethral valves. Magnetic resonance imaging (MRI) of the thoracic and lumbar spine (Figure 3) was conducted to exclude a neurological aetiology. This demonstrated no features of spinal dysraphism or spinal cord abnormality; however, bilateral hydronephrosis was evident as documented on ultrasound. Incidentally, the MRI also demonstrated a homogenous cystic lesion within the prostate gland (Figure 4) with mass effect on the bladder base. Only limited sequences through the prostate were obtained and contrast was not administered. Given the background of failure to respond to medical treatment and the progressive symptoms, a PA was considered as the likely aetiology for the obstructive uropathy.

The patient underwent drainage of the abscess. *Staphylococcus aureus* was cultured and intravenous antibiotics were administered. The blood cultures and nasal swabs excluded haematogenous and distant focus of infection. Improvement in symptoms was noted following drainage of the abscess. On follow-up, the patient demonstrated a clinical response to treatment with resolution of the symptoms. Follow-up sonographic images were not available as they were performed at another institution, however the clinician documented resolution of the hydronephrosis.

Paediatric prostatic abscess is an uncommon condition and continues to remain a difficult clinical diagnosis to consider. In this case, it was only considered as an incidental finding on the limited images through the bladder base. This highlights the importance of considering PA formation in paediatric patients who present with signs and symptoms of renal tract infection and bladder outlet obstruction. Early diagnosis and treatment of the condition has significant benefits in terms of the relief of patient symptoms and prevents further damage to the renal tracts.

### Ethical considerations

This article followed all ethical standards for research. Data and images in the manuscript were anonymised.

## Discussion

Urinary tract obstruction can result from congenital or acquired lesions that may occur at any point from the calyx to the tip of the urethra. Most childhood obstructive lesions are congenital.^4^ In the presence of bilateral hydronephrosis, vesicoureteral reflux and bladder outlet obstruction are the most likely causes. The common causes of bladder outlet obstruction may be divided according to the level of the obstruction. At the level of the bladder, bladder neck hypertrophy and neuropathic bladder may be considered.^4^ Urethral valves, strictures and polyps are common causes for the urethral cause of obstruction.^4^ Prostatic abscess with mass effect and obstructive uropathy represents a rare cause of obstructive uropathy. Other causes of cystic prostate lesions in the paediatric age group include a prostatic utricle cyst and a Müllerian duct cyst.^5^

Prostatic abscess is a rare clinical entity and typically presents in the fifth to sixth decade of life. The diagnosis is often challenging as it mimics a lower urinary tract infection.^6^ The symptoms include dysuria, increase in frequency, fever, urinary retention and leucocytosis.^6^ The persistence of the symptoms despite antibiotic treatment should alert the clinician to the possibility of a PA.^6^

In the background of a low prevalence of the clinical entity, as well as the wide range of local and systemic symptoms, it is imperative to understand the risk factors and maintain a high index of suspicion within these patient profiles. In adults, diabetes mellitus has been identified as the most common co-morbidity, with a reported co-prevalence of 63%.^1^ Other causes of immunosuppression including human immunodeficiency virus (HIV), liver cirrhosis, renal failure and haemodialysis have been cited as predisposing factors for prostatic abscess.^1^ Chemotherapy, organ transplant or previous genitourinary procedures also present a higher risk of prostatic abscess.^3^

Two mechanisms for the pathogenesis of prostatic abscess have been described. The first is via the retrograde flow of contaminated urine through the prostatic duct and subsequent infection of the prostate. Bladder outflow obstruction and immunosuppression are predisposing factors and enteric organisms such as *Escherichia coli* and other coliform bacteria are the commonest causative organisms. The less common mechanism of infection is secondary to haematogenous spread from a distant focus. This includes a variety of organisms, including *S. aureus, Mycobacterium tuberculosis, E. coli, Klebsiella pneumoniae, Pseudomonas aeruginosa, Burkholderia pseudomallei* and *Candida* spp.^1^

The symptoms overlap with symptoms of other lower urinary tract pathology, and the diagnosis of a prostatic abscess is often delayed. Hence it is often only diagnosed after the failure to respond to medical treatment.^1^ The diagnosis may be difficult because flocculence is present in only one third of patients. In addition, rectal examinations are not routine for infants.^7^ More than 90% of patients will present with a leucocytosis and almost all patients will demonstrate a leukocyturia.^8^

A prostatic abscess may complicate, fistulate and drain into one of the surrounding pelvic structures. Abscesses located at the base of the prostate gland commonly fistulate to the bladder or prostatic urethra, and those near the apex result in a rectal or perianal fistula. Severe cases of a prostatic abscess may extend into the seminal vesicle and spermatic cord.^1^

In the paediatric population, ultrasound is the initial diagnostic modality of choice to evaluate the paediatric lower urinary tract. A 5-megahertz (MHz) to 7-MHz transducer is used transabdominally and, when high resolution is required, a 7-MHz to 12-MHz transducer may be used transperineally. The paediatric prostate is visualised through a full bladder as a hypoechoic, elliptically shaped, soft tissue structure at the bladder base.^9^ A prostatic abscess appears as a hypoechoic or anechoic lesion with more or less defined edges and a peripheral hyperechoic halo.^10^ They are often of varying sizes, contain internal septations and are typically located in the transitional and central zones.^3^

Magnetic resonance imaging defines the cross-sectional anatomy of the prostate with greater tissue differentiation compared to computed tomography (CT). The prostate shows homogenous signal intensity on MRI imaging that is isointense to muscle on T1-weighted images and iso- to hyper-intense to muscle on T2-weighted images before puberty. After puberty, the signal characteristics resemble those of the adult prostate.^9^ A prostate abscess appears hypointense on T1-weighted MRI and hyper-intense on T2-weighted images.^1^

Reports of prostatic abscesses in neonates have been published. Mann described three cases of acute staphylococcal prostatitis causing urinary obstruction in neonates.^11^ Collins et al.^7^ reviewed 13 cases of neonatal prostatic abscess and described *Staphylococcus* spp. in 77% (10 out of 13) of the reviewed cases. *Staphylococcus* spp. is a rare cause of paediatric urinary tract infection, suggesting that paediatric prostatic infection likely arises from haematogenous spread rather than an ascending urinary tract infection.^6,7^ Chao and Yang^12^ described a case of a large prostatic abscess in an adolescent successfully treated with antibiotics. Prior to this, only three cases of prostatic abscess in adolescents have been reported.^12^

Reports of prostatic abscess secondary to *S. aureus*, specifically methicillin resistant *S. aureus* (MRSA), have increased recently.^13^ Kiehl et al.^6^ reported the case of a 15-year-old male with an MRSA prostatic abscess. In that case, the patient was noted to have a history of MRSA abscesses and recent hospitalisation.^6^ Foster et al.^13^ documented the first reported case of methicillin-susceptible *S. aureus* (MSSA) prostatic abscess in a paediatric patient beyond the neonatal period.

Prostatic abscess in the paediatric population is a rare occurrence. The referenced cases and our case report highlight the importance of a systematic radiological analysis of the upper and lower urinary tracts in a patient presenting with bilateral obstructive uropathy. Interestingly, our index patient did not demonstrate haematogenous spread or a distant focus of infection and responded completely to medical and surgical intervention. Upon exclusion of the common causes for bilateral obstructive uropathy, a high level of clinical suspicion is needed to diagnose this entity, which in the paediatric population likely arises secondary to haematogenous spread.^13^

## Conclusion

Although rare, few cases of paediatric PA have been reported in neonates and the finding of a PA in a 6-year-old is an even rarer event. The few case reports describing this clinical entity suggest that prostate infection likely arises from haematogenous spread. Prostatic abscess represents a diagnostic and therapeutic challenge, and in the systematic evaluation of an obstructive or non-obstructive aetiology of bilateral hydronephrosis, it is imperative to consider a PA or prostatic lesion as a possible aetiology, as it is often overlooked because of its rarity.
